# Onset of Celiac Disease after Treatment of Chronic Hepatitis C with Interferon Based Triple Therapy

**DOI:** 10.1155/2015/763497

**Published:** 2015-11-18

**Authors:** Amandeep Singh, Nayere Zaeri, Immanuel K. Ho

**Affiliations:** ^1^Crozer Chester Medical Center, One Medical Center Boulevard, Upland, PA 19018, USA; ^2^Cleveland Clinic Foundation, 9500 Euclid Avenue, Cleveland, OH 44195, USA; ^3^Division of Gastroenterology, Pennsylvania Hospital, 230 W. Washington Square, 4th Floor, Philadelphia, PA 19106, USA

## Abstract

*Background*. Patients treated with interferon (IFN) based therapies may develop exacerbation of autoimmune disease. We herein present the case of a 53-year-old female patient who developed celiac disease (CD) as a result of triple therapy (interferon, ribavirin, and boceprevir) for chronic HCV.* Case*. 53-year-old Caucasian female with past medical history of IV drug abuse was referred for abnormal LFTs. Laboratory data showed HCV RNA of 4,515,392 IU/mL, HCV genotype 1a, with normal LFTs. She was treated with 4 weeks of pegylated interferon alfa-2a plus ribavirin, followed by triple therapy using boceprevir for a total of 28 weeks. Approximately 4 weeks after initiation of triple therapy patient developed loose nonbloody bowel movements and was also found to have anemia. Biopsies from first and second portions of the duodenum were consistent with CD. The patient was treated with a gluten-free diet. Her intestinal symptoms improved and the hemoglobin returned to normal.* Conclusion*. Chronic HCV patients being treated with interferon alfa can develop celiac disease during or after therapy. For patients with positive autoantibodies, all-oral-IFN-free regimens should be considered. Celiac disease should be considered in patients who develop CD-like symptoms while on and shortly after cessation of interferon alfa therapy.

## 1. Introduction

Chronic hepatitis C (HCV) is one of the most common causes for liver transplantation. There are at least 5.2 million persons living with chronic HCV in the United States today [1]. The standard of care for treatment of chronic HCV genotype 1 infection before the approval of newer interferon-free regimens was a triple combination regimen therapy involving pegylated interferon alfa, ribavirin, and a NS3/4A protease inhibitor (boceprevir, telaprevir, and simeprevir) or a nucleotide analogue NS5B polymerase inhibitor (sofosbuvir) or peginterferon and ribavirin alone. Some patients may develop autoimmune side effects related to underlying disorders while on interferon treatment [2]. We herein present a case of a 53-year-old female who was started on triple therapy (interferon, ribavirin, and boceprevir) for HCV and subsequently developed celiac disease (CD) as a result of therapy.

## 2. Case Description

C. K., a 53-year-old Caucasian female, was referred by her primary care physician for abnormal liver function tests. She was first diagnosed with HCV exposure in 2005 with genotype 3a and had undergone pegylated interferon monotherapy weekly for six months. She achieved end of treatment response. Subsequently, she continued to use intravenous drugs. On presentation to our clinic on 05/04/2011, patient was found to have reinfection with genotype 1a with an HCV viral load of 29,000 IU/mL. She had no nausea, vomiting, heartburn, abdominal pain, or dysphagia. She had lost some weight but she was dieting. She denied any intravenous drug and alcohol use in last 3 years.

Her past medical history was notable for methicillin resistant staphylococcus aureus (MRSA) endocarditis, vertebral osteomyelitis, hyperlipidemia, diverticulosis, and* Helicobacter pylori* gastritis. There was no family history of celiac disease, type 1 diabetes or any other autoimmune diseases. She is a former smoker. Her medications included butalbital/acetaminophen/caffeine, simvastatin, and esomeprazole.

Initial physical examination showed temperature of 98.2°F, pulse of 72 beats/minute, BP of 116/70 mm/Hg, and weight of 146 pounds. Physical examination was normal “without stigmata of liver disease or malnutrition and so forth.”

**Figure 1:**
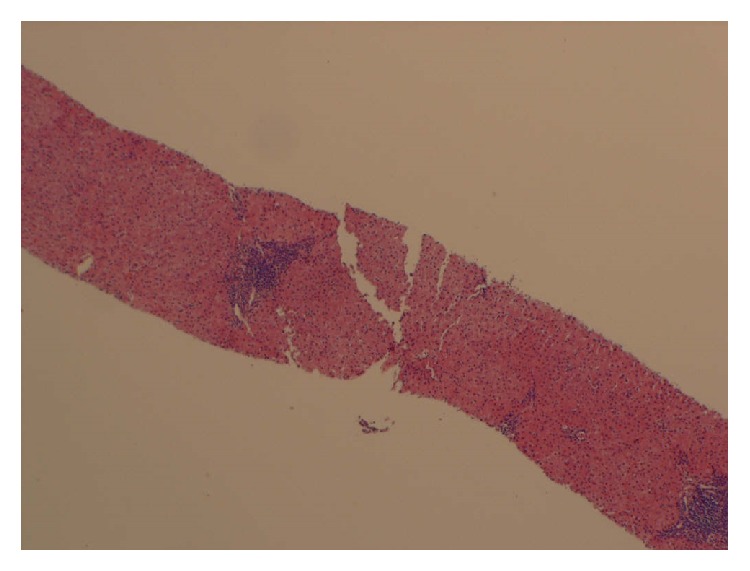
Liver biopsy showing grade 2 inflammation activity and stage 1 fibrosis.

Laboratory data on initial visit showed normal complete blood count (CBC), complete metabolic profile (CMP), and iron studies. Pt was found to have HCV viral load of 4,515,392 IU/ml and genotype 1a. Other causes of liver disease including hepatitis B, autoimmune hepatitis, Wilson disease, and HIV were ruled out. The patient underwent a liver biopsy which showed Metavir grade 2 inflammatory activity and stage 1 fibrosis (Figure 1).

Colonoscopy was also done on 6/25/2008 at different facility, which did not show any polyps but showed diverticular disease of descending colon and sigmoid colon. The patient underwent upper gastrointestinal endoscopy (EGD) done on 10/7/2009 which was unremarkable.

Treatment was initiated on dual therapy with pegylated interferon alfa-2a at 180 *μ*g subcutaneous weekly plus ribavirin 600 mg orally twice per day for a 4-week lead-in phase, followed by triple therapy using boceprevir (BOC) 800 mg orally three times per day for total of 28 weeks. HCV viral loads which were collected at weeks 4, 8, 12, 21, and 24 were undetectable by polymerase chain reaction. During treatment, the patient developed neutropenia and anemia, which were treated with pegfilgrastim and darbepoetin alfa.

Approximately 4 weeks after initiation of triple therapy the patient developed loose nonbloody bowel movements, which was not reported at the time. She experienced nausea and decreased appetite but no vomiting or abdominal pain. Occasionally she felt bloated. There was no fever, chills, or skin rash.

**Figure 2:**
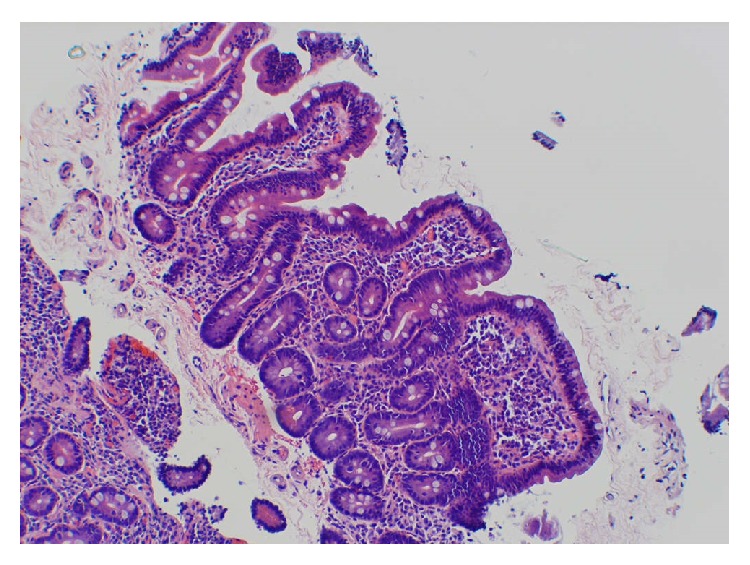
Duodenal biopsy showing mild villous blunting, crypt hyperplasia, and intraepithelial lymphocytes characteristic of celiac disease.

Three months following cessation of treatment the patient complained of ongoing weakness and dyspnea on exertion which had begun after the initiation of therapy but which had not resolved completely. She had ten-pound weight loss during treatment which persisted, but she also now admitted to having 5-6 watery loose bowel movements for the preceding five months. Repeat CBC showed low hemoglobin of 10.7 g/dL (baseline 14 g/dL). The patient failed to give stool samples for analysis. She underwent an EGD and colonoscopy. During EGD, mucosa in the stomach and second and third part of duodenum appeared normal but biopsies from second portion of the duodenum revealed mild villous blunting, mild crypt hyperplasia, and minimal intraepithelial lymphocytes consistent with CD (Figure 2). The tissue transglutaminase IgA was 85 U/mL. The patient was started on gluten-free diet. Her intestinal symptoms improved and the hemoglobin returned to normal.

## 3. Discussion

Development of CD has been reported in patients with chronic hepatitis C [3, 4] and CD activation after the initiation of interferon alfa has also been described [5–8]. There are studies which deny any clear association or increased prevalence of CD in patients with HCV [9–11] but, on the contrary there are studies to support the increased prevalence of celiac autoantibodies in patients with chronic hepatitis C and risk of activation of silent CD during interferon treatment [7, 8].

Autoimmune side effects including hyperthyroidism, hypothyroidism, diabetes mellitus, interstitial pneumonitis, autoimmune thrombocytopenic purpura, hemolytic anemia, rheumatoid arthritis, and systemic lupus erythematosus have all been reported to exacerbate or develop during interferon therapy [2].

CD is an autoimmune disease prevalent in Caucasians of European descent (1 : 200–300) and is mediated by CD4 lymphocytes in response to ingested gluten in genetically predisposed individuals. Gama interferon production, stimulated by gluten, activates CD4 lymphocytes in lamina propria of small intestine mucosa resulting in intestinal mucosa damage with villous atrophy, crypts hyperplasia; with mucosa infiltration with CD3^+^ lymphocytes [6]. CD may present in various clinical modes such as potential, latent, silent, atypical, and classical modes.

Interferon alfa and ribavirin treatment for chronic HCV may unmask the symptoms of celiac disease [5–8]. However, diarrhea, the hallmark symptom of celiac disease, may also occur as a result of IFN-alpha therapy [12]. Interferon use has the potential to exacerbate autoimmune disease either by direct effects on tissues or through its effect on the immune system by altering lymphocyte population and the profile of cytokine production. While interferon alfa acts in differentiation of T helper (Th) 2 cells to Th1 cells and improve T-cell and natural killer cell cytotoxicity [6], ribavirin promotes a Th1 cytokine-mediated immune response, suppressing Th2 response [13]. Similarly, gluten induced activation of lamina propria Th1 cells followed by secretion of IFN gamma is an important pathogenic mechanism in CD [14]. Thus one might speculate that Th1/Th2 imbalance may play a role in the activation of CD in some patients. Our patient was on boceprevir (BOC), along with IFN and ribavirin.

This is a case of patient manifesting celiac disease on and after treatment with interferon based triple regimen. Whether BOC has additive effects on IFN-ribavirin is unknown. Our experience with this case raises again the important question of whether CD screening should be performed prior to initiation of interferon based therapy for chronic hepatitis C [15]. Although screening for celiac disease before initiation of interferon therapy has been suggested [16, 17], current screening recommendations for celiac disease are targeted towards pediatric and adult patients already diagnosed with certain autoimmune diseases (including autoimmune thyroid disease, autoimmune liver disease, primary biliary cirrhosis, and type 1 diabetes mellitus) [18, 19]. Screening for CD in the setting of HCV treatment with interferon has not yet been recommended. Patients with positive autoantibodies require careful consideration of IFN-free regimens. If IFN-free regimens are not available there should be a low threshold for obtaining tissue transglutaminase IgA from patients if diarrhea, weight loss, or anemia developed while on or after the use the interferon therapy and if the suspicion of CD is high, intestinal biopsy should be pursued even if serologies are negative and a gluten-free diet must be started preemptively.

## 4. Conclusion

Chronic HCV patients being treated with interferon alfa can develop celiac disease during or after therapy. For patients with positive autoantibodies, all-oral-IFN-free regimens should be considered. Celiac disease should be considered in patients who develop CD-like symptoms while on and shortly after cessation of interferon alfa therapy.
